# Superb Monocular Depth Estimation Based on Transfer Learning and Surface Normal Guidance

**DOI:** 10.3390/s20174856

**Published:** 2020-08-27

**Authors:** Kang Huang, Xingtian Qu, Shouqian Chen, Zhen Chen, Wang Zhang, Haogang Qi, Fengshang Zhao

**Affiliations:** 1 Department of Mechanical Engineering and Automation, School of Mechanical and Aerospace Engineering, Jilin University, Changchun 130022, China; huangkang18@mails.jlu.edu.cn (K.H.); quxt@jlu.edu.cn (X.Q.); zhenchen18@mails.jlu.edu.cn (Z.C.); qihg18@mails.jlu.edu.cn (H.Q.); 15951264800@163.com (F.Z.); 2 Research Center for Space Optical Engineering, Harbin Institute of Technology, P.O. Box 307, Harbin 150001, China; shouqian.chen@hit.edu.cn

**Keywords:** SLAM, SFM, supervised deep learning, multi-task learning, transfer learning, monocular depth estimation, surface normal estimation

## Abstract

Accurately sensing the surrounding 3D scene is indispensable for drones or robots to execute path planning and navigation. In this paper, a novel monocular depth estimation method was proposed that primarily utilizes a lighter-weight Convolutional Neural Network (CNN) structure for coarse depth prediction and then refines the coarse depth images by combining surface normal guidance. Specifically, the coarse depth prediction network is designed as pre-trained encoder–decoder architecture for describing the 3D structure. When it comes to surface normal estimation, the deep learning network was designed as a two-stream encoder–decoder structure, which hierarchically merges red-green-blue-depth (RGB-D) images for capturing more accurate geometric boundaries. Relying on fewer network parameters and simpler learning structure, better detailed depth maps are produced than the existing states. Moreover, 3D point cloud maps reconstructed from depth prediction images confirm that our framework can be conveniently adopted as components of a monocular simultaneous localization and mapping (SLAM) paradigm.

## 1. Introduction

### 1.1. Background

Compared with depth estimation methods relying on laser rangefinders or other optical instruments, the computer vision method does not require expensive optical equipment and repeated lens calibration. Therefore, image-based depth prediction has been extensively studied and widely applied to 3D scene understanding tasks, such as structure from motion (SFM) [1,2], simultaneous localization and mapping (SLAM) [3,4], 3D object detection [5], etc.

The computer vision method, i.e., image-based depth estimation, defines image depth as the distance from the object point corresponding to each pixel to the camera and exploits clues of images like linear perspective, focus, occlusion, texture, shadow, gradient, etc. for calculation. All the image-based methods can be summarized as two classes: stereo vision methods and monocular methods. The stereo vision methods are heavily dependent on natural light in a natural environment to collect images and is sensitive to changes in illumination angle and changes in illumination intensity. The differences in image matching of the two pictures will result in considerable differences from the matching algorithm. Compared with the stereo vision methods, the monocular vision systems [6,7,8,9] rarely encounter the aforementioned problems. Therefore, the monocular depth estimation method is more convenient for calibration or identification than the stereo vision approach.

Despite great achievements [6,10,11] in terms of monocular depth estimation, previous research has not given it enough attention [6,7,10,11,12]. First, not enough importance is attached to intrinsically consistency such as semantics labels, color clues of the red-green-blue (RGB) images, the surface normal of the depth images, and the linear perspective of the depth images. The state-of-the-art (SOTA) algorithms [7,9,11,13] leverage fairly complex deep neural networks that are too slow for real-time inference.

### 1.2. Ideas

In terms of the first content above, there are several methods for acquiring image depth based on supervised learning that can achieve ideal depth maps. However, establishing labeled datasets such as NYU depth V2 [14], Scan-Net [15], and Make-3D [16] is such a challenge for supervised learning methods, such that the datasets [14,15,16] have been constructed for over five years. The ground truth (GT) depth images from these datasets were thus captured by the outdated depth sensors. Therefore, the geometric accuracy of GT images definitely fluctuated, which would cause side effects on the precision of both training and testing processes. Therefore, we first predicted depth maps from RGB images based on an encoder–decoder structure and denoted those images as “coarse depth maps.” Then, surface normal maps were estimated and used for applying geometric guidance to the depth estimation process, i.e., the surface normal estimation was introduced in this paper so as to enable accurate regression of geometric structure and complex boundaries of 3D objects.

As for the second content, previous authors [6,17,18] chose to propose a lighter-weight model-based encoder–decoder network, which produced more accurate depth estimation maps and reduced computational complexity. Wofk et al. [17] demonstrated that a well-designed low latency network can maintain real-time depth estimation with economical computation costs. Alhashim et al. [6] proved that a very simple transfer learning-based decoder robustly achieves high-resolution depth maps. Previous studies [6,9,19] proved that the Dense-net is more suitable for depth estimation than models like SE-Net, Res-Net, and Mobile-net. However, experiments in references [6,9] showed that pre-trained deep learning structures based on Densenet-161, Densenet-169, and Densenet-201 models cannot afford real-time depth estimation. As a result, a lighter-weight depth estimation network Densenet-121 was adopted in this paper, which requires fewer parameters and inference iterations than other dense-net models. Moreover, in order to enhance the performance of the Densenet-121 model, the surface normal maps were jointly estimated with depth images.

### 1.3. Approach

The reform of current monocular depth framework can be summarized as follows. First, a novel depth estimation network called the coarse depth estimation (CDE) network was proposed, which leverages an efficient encoder–decoder network architecture. In terms of deep-feature-encoding layers, we introduced a pre-trained Densenet-121 model that was primitively proposed for image classification and object detection. As for the decoder, we chose the 2× bilinear proposed by Alhashim et al. [6] as the up-sampling method. Second, we put forward a network called the red-green-blue-depth (RGB-D) surface normal (RSN) network in order to obtain more accurate surface normal maps. The RSN network was designed as a two-stream encoder–decoder architecture, which produced surface normal maps by hierarchically fusing coarse depth produced by the CDE network and RGB images from a selected dataset. Then, the refinement network exploited surface normal maps produced by the RSN network for ameliorating the quality of coarse depth images.

Three-dimensional features can be represented with different formats, including point clouds, meshes, and volumetric grids. Specifically, the point cloud map is a set of data points preserving the original geometric information of 3D objects without any discretization. Three-dimensional point cloud maps were used as a criterion for evaluating the depth maps produced in our study.

### 1.4. Contributions

**Figure 1:**
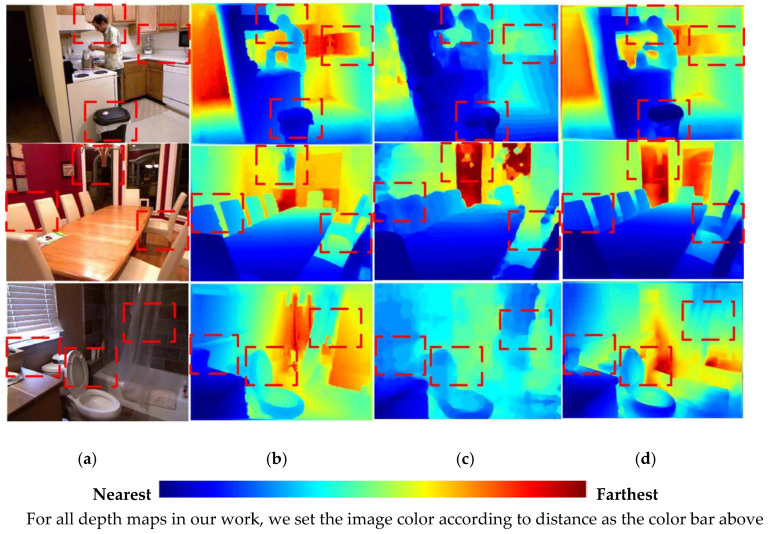
The comparison of depth maps were produced by different methods. (**a**) Raw red-green-blue (RGB) images (**b**) Ground truth (GT) depth maps [14], (**c**) Depth maps from the state-of-the-art (SOTA) practice [7], (**d**) Depth maps from our depth prediction network.

Different from previous studies [10,20], we remodeled the conventional depth estimation network layout. Repeated experiments proved that a deep network based on the low latency Densenet-121 model affords real-time coarse depth estimation and depth refinement depending on a Tesla M40 produced by NVIDA in Santa Clara, CA, U.S. with single 12G memory capacity. Benefiting from guidance of surface normal maps, the Densenet-121 based network obtained better depth maps than SOTA practices [6,7,9]. As shown in Figure 1, our network outperforms SOTA depth estimation on an NYU [14] dataset and produces higher-resolution results that capture object boundaries more faithfully.

**Figure 2:**
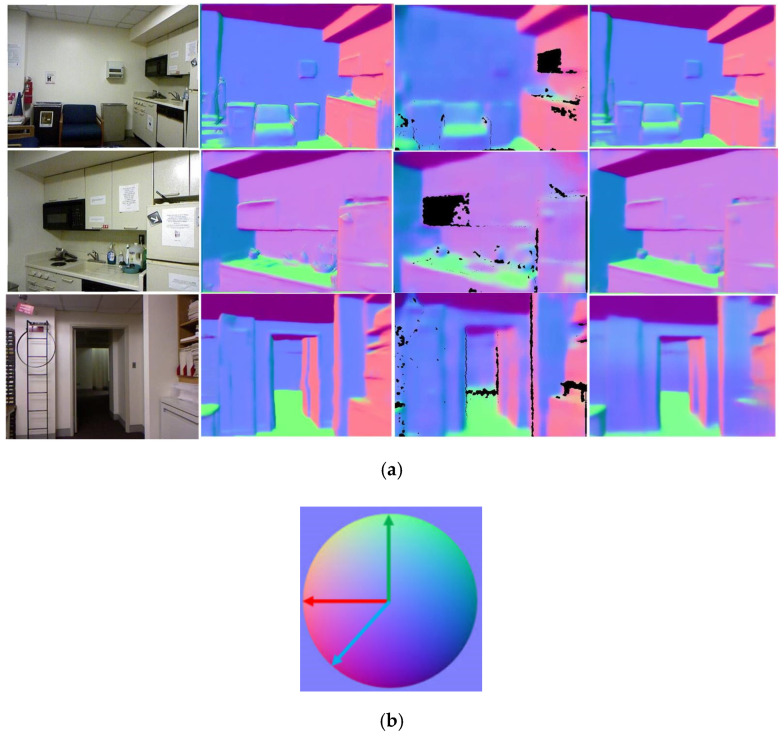
The comparison of surface normal maps: (**a**) from left to right: RGB images, Ground-Truth (GT), surface normal maps produced by Qi et al. [10], ours. (**b**) Color-map definition: red represents left, green represents up, and blue represents outward.

Our second contribution is reflected in proposing an RGB-D surface normal network, which effectively captures the geometric relationships between RGB and depth images. Different from previous frameworks [21,22], we proposed a fusion network leveraging both RGB and coarse depth prediction images instead of using RGB images only. Images from different domains can complement each other in the surface normal estimation process, i.e., coarse depth images from the CDE network contribute to enhance geometry details, and RGB images make up the missing depth pixels. As shown in Figure 2, we achieved better surface normal maps than Qi et al. [10] based on the RGB-D surface normal network. Moreover, as we can see in Figure 1, with the geometrical guidance of surface normal, depth maps with distinct boundaries were acquired. 

## 2. Related Work

Stereo depth estimation can be regarded as a well-posed process once ignoring the problem of occlusions and depth discontinuities. Moreover, depth estimation methods based on stereo vision achieved even more accurate and robust depth maps than RGB-D sensors [14,15]. Meanwhile, more precise depth features from our monocular method can also make contributions to better multi-view stereo reconstruction.

Monocular depth estimation has been considered by plenty of researchers [23,24,25,26,27,28,29], which usually defined the estimation as a regression of the depth map from a single RGB image. Eigen et al. [21] introduced the application of CNNs in monocular depth estimation, which inspired researchers to explore methods based on deep learning. At present, deep learning methods play a leading role in monocular depth estimation practices. Generally, deep learning methods can be divided into supervised approaches, self-supervised approaches, and unsupervised approaches. Supervised monocular depth methods achieved great breakthroughs relying on well-annotated ground-truth images offered by datasets [14,15,16]. For example, Liu et al. [28] combined CNNs with CRF to learn the super-pixel-wise connections between depth and RGB images. Different from the supervised method, self-supervised methods usually set up a separate camera pose estimation network [26,27] or a jointly calculated optical flow and camera pose [30]. Unsupervised practices learn the scene depth image synthesis [10] or ego-motion in monocular videos without using ground truth data [24,25]. Similar to the SOTA methods [2,7], in recent years, the supervised learning method was selected in this paper.

Multi-task/cross-task learning was designed based on intrinsic connections of physical elements of the research targets selected. Some recent works attempt to investigate the sharing of image features between different tasks [31,32,33]. Jiao et al. [34] jointly trained semantic labeling and depth estimation in their encoder–decoder network architecture. Zhang et al. [11] proposed a pattern-affinity propagation method for jointly predicting depth, surface normal and semantic segmentation. As for us, the proposed network first jointly predicts depth and surface normal and then takes advantage of surface normal maps to refine the predicted coarse depth maps.

Surface normal guidance has been introduced by previous studies [10,19,20], where they employed surface normal maps as 3D cues for improving the geometric quality of monocular depth images. Qi et al. [10] jointly calculated depth and surface normal from a single image, making the final estimation geometrically more precise. In work of Zeng et al. [19], a skip-connected architecture was proposed to fuse features from different layers for surface normal estimation. A novel 3D geometric feature virtual normal was proposed by Yin et al. [20] to refine the predicted depth maps. Surface normal estimation was adopted in this paper for calculating the angular difference between predicted depth images and ground-truth maps, which thus applied geometric restriction to depth images.

A transfer-learning-based deep framework was adopted by Zhang et al. [11] to obtain the state-of-the-art semantic segmentation maps. We adopt a pre-trained Densenet-121 model [5] as the backbone of the coarse depth estimation network in depth feature extraction. Our method benefits from the application of transfer learning, where we take advantage of encoders originally designed for 3D object detection by Alhashim [6].

Encoder–decoder networks were widely adopted in various computer vision tasks such as image classification, image segmentation, and 3D objects detection. In recent years, such architectures made significant contributions to both supervised learning and unsupervised learning-based practices of monocular depth estimation [3,30]. We devised a compendious but effective symmetrical encoder–decoder structure with skip connections. Repeated experiments indicated that our encoder–decoder network with simple structure can outperform the SOTA depth synthesis based on more complicated deep learning architecture [7,9].

## 3. Our Method

This section presents our method for monocular depth estimation. First, the general deep learning framework is introduced. Then, rational loss functions for overall training process are defined. Finally, we discuss the practice of 3D point cloud maps reconstruction.

### 3.1. Framework Overview

**Figure 3:**
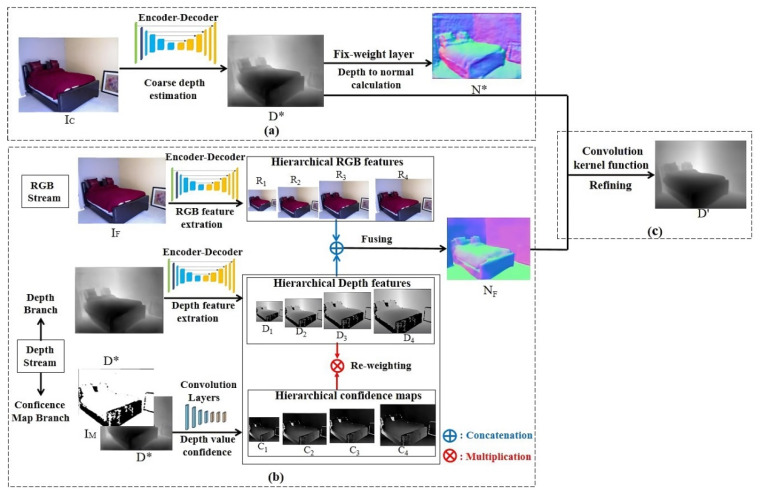
General estimation framework. (**a**) Coarse depth estimation network; (**b**) red-green-blue-depth (RGB-D) surface normal network; (**c**) refinement network.

As shown in Figure 3, the whole deep learning structure consists of three parts: a coarse depth estimation network, an RGB-D surface normal network, and a refinement network. 

The coarse depth estimation (CDE) network leverages efficient encoder–decoder network architecture. Coarse depth images generated from the CDE network are then fed to the RGB-D surface normal network for RGB-D fusion. Moreover, coarse depth images are also converted to coarse surface normal maps based on a fix-weight network [4]. For convenience, we describe a single RGB input image as$\;I_{C}$, a single in-painted ground truth depth image produced by Levin [35] as$\;D^{GT}$, an output coarse depth map as $D^{*}$, and a coarse surface normal map recovered from $D^{*}\;$as$\;N^{*}$. 

The RGB-D surface normal (RSN) network was designed for obtaining accurate surface normal maps, which functions as the refining coarse depth map ($D^{*}$). As shown in Figure 3, the RSN network can be divided into two streams—the RGB stream and the depth stream. The latter can be further divided into depth branch and the confidence map branch. For the general architecture of RSN network, we define a single RGB input as$\;I_{F}$, a corresponding coarse depth input as$\;D^{*}$, and a surface normal output map as$\;N_{F}$. 

The RGB stream and depth branch in Figure 3 operate respectively to generate RGB feature$\;R_{1}\sim \;R_{4}$ and relative sensor depth feature$\;D_{1}\sim \;D_{4}$ with hierarchical resolution. Then, the two branches cooperate to combine and fuse features from each branch.

The ground truth depth images ($D^{GT}$) used for training the CDE network were produced following Levin et al. [35]. This method greatly contributes to fill pixel holes in sensor depth images from NYU-Depth-V2 [14]. However, this method cannot eliminate the side effects caused by lost pixels on accuracy of ground truth depth images and corresponding coarse depth images ($D^{*})$. Therefore, a confidence map network branch was set following the method proposed by Zeng et al. [11], which generates confidence maps to indicate whether side effects resulted from pixel holes on$\;D^{*}\;$or not. Confidence maps [19] of depth image were produced by combining mask images [21] ($I_{M}$) with relative coarse depth images ($D^{*})$ and were denoted as$\;C_{1}\sim \;C_{4}$ according to resolution. 

The refinement network actually servers as the convolution kernel function, which optimizes the coarse depth maps from the CDE network guided by surface normal maps from the RSN network. Finally, with the aid of the refinement network, a superbly accurate depth map$\;D^{\prime }$ was generated. 

### 3.2. Coarse Depth Estimation (CDE) Network

**Figure 4:**
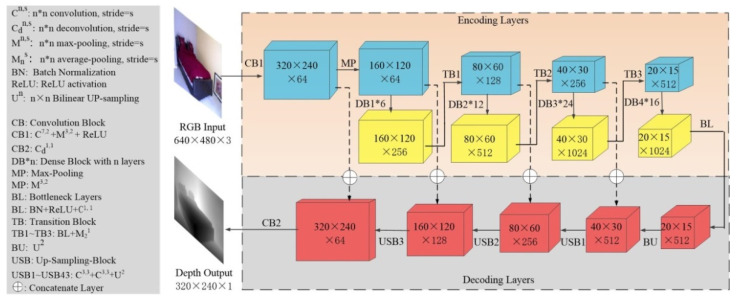
Encoder–decoder coarse depth network.

Figure 4 shows the distinct structure of the encoder–decoder network for getting coarse depth maps ($D^{*}$). For the encoder, the raw RGB image ($I_{C}$) is encoded into a feature vector based on a Densenet-121 [5] model that has been pre-trained on Image-Net [36]. The feature vector is then transmitted to a sequence of up-sampling layers to produce $D^{*}$ with half resolution of$\;I_{C}$. As for decoding operation, the decoder network consists of four up-sampling units (${\mathrm{BU}}_{1}\;$and${\;\mathrm{USB}}_{1}\sim {\mathrm{USB}}_{3}$) and relative concatenation (⨁) skip-connections. In decoding layers, the 2× bi-linear interpolation proposed by Alhashim [6] is adopted as an up-sampling method. 

**Figure 5:**
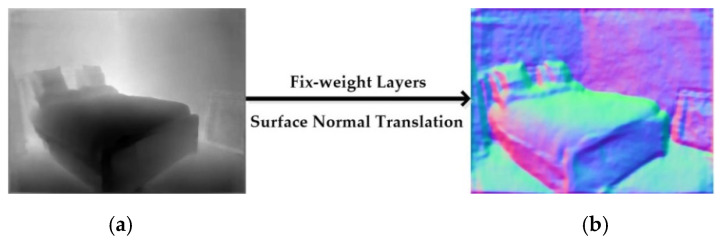
Generating coarse surface normal image. (**a**) Coarse depth ($D^{*}$); (**b**)$\;N^{*}$.

As shown in Figure 5, the coarse image $D^{*}$ is then converted to coarse surface normal image ($N^{*}$) based on least square algorithm [37], and the inference network is just a fix-weight network [11]. 

### 3.3. RGB-D Surface Normal (RSN) Network

**Figure 6:**
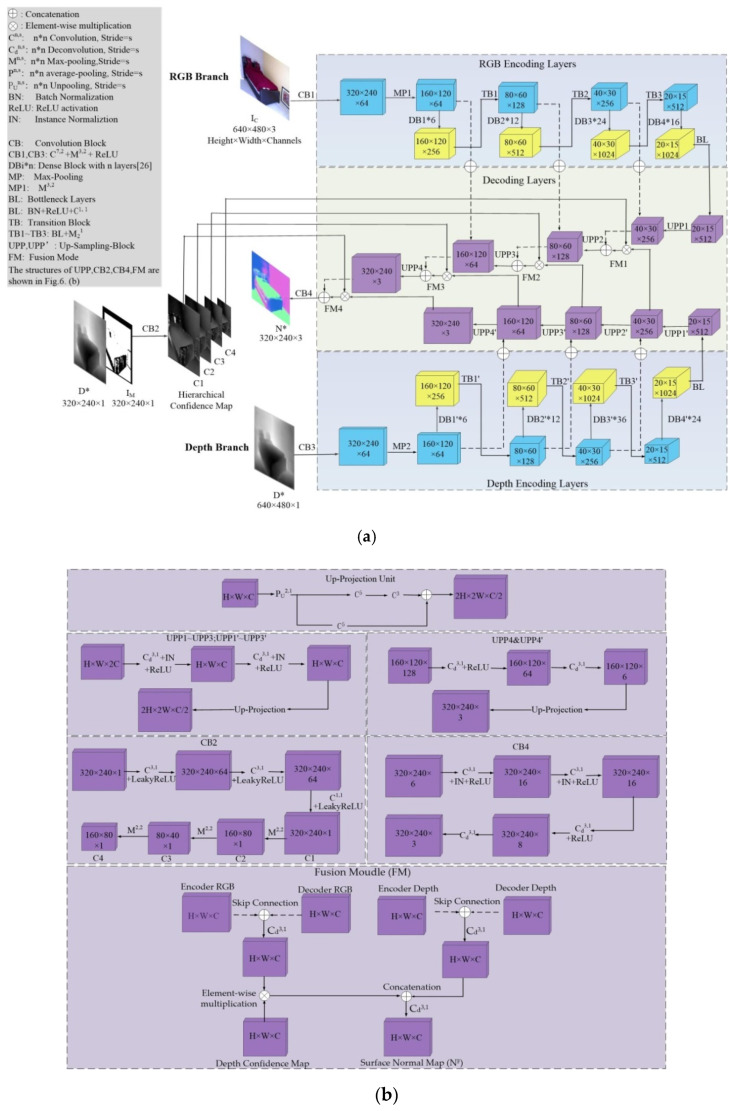
Surface normal adjustment network (Dense-net-121 based). (**a**) The general structure of the RGB-D surface normal network (RSN) network; (**b**) the architectures of up-projection units, fusion module and convolution blocks.

Both the RGB stream and depth branch leverage encoder–decoder network architecture. The two branches employ a pre-trained Densenet-121 model as encoding backbone [26], whose detailed structure is illustrated in the upper row of Figure 6. Generally, the encoder consists of same convolution layers with the raw Densenet-121 [5] encoder, except the last convolution blocks. The number of channels was reduced from 1024 to 512 via bottleneck layers aiming to reduce redundant parameters. A symmetric decoder equipped with concatenation connections for the refitted encoder was designed. Multi-scale up-sampling layers were introduced to decoder, which enables RGB-D images fuse in different scales. What’s more, common pooling masks were set to let the network learn more image features.

Different from CDE Network, the RSN Network leverages coarse depth images ($D^{*}$) instead of in-painted [35] ground truth depth images ($D^{*}$) as the depth input. The pre-trained RSN Network can thus be applied for estimating surface normal maps based on custom images. 

As is shown in Figure 3, pixel holes in mask images ($I_{M}$) suggest that there are lots of missing pixels in ground truth depth images, which inevitably causes deviation to supervised learning. Therefore, we adopted a multi-layer convolution network (CB2) for producing confidence map $C_{l}$ [19] of input depth images. l stands for scale value of images, i.e., if the resolution of 2D images can be denoted as H×W, then the corresponding l is defined as {(l,H×W)}={(1,40×30);(2,80×60);(3,160×120);(4,320×240)}. The detailed structure of CB2 is shown in Figure 6b. The depth branch also adopts Densenet-121 model–based encoding layers, and the fusion calculation takes place at the decoder side. As shown in Figure 6a, the depth features$\;D_{l}^{*}\;$are passed into the fusion module in each scale l and re-weighted with the confidence map$\;C_{1}\sim \;C_{4}$. Then re-weighted$\;D_{l}^{*}\;$are concatenated (⨂) with color features with same resolution and transmitted to a de-convolution layer to produce surface maps based on RGB-D fusion. Finally, the convolution block (CB4) of RSN Network generates the surface map$\;N^{*}$. The RGB-D fusion algorithm [19] can be expressed as Equation (1).

(1) $$N_{l}^{\prime }=\;deconv[I_{F_{l}}⨁\left(C_{l}\;⨂\;D_{l}^{*}\right)]$$

The decoder layers of the RSN network were also designed based on the Densenet-121 model. Different from the CDE network, up-projection units [17] (${\mathrm{UPP}}_{1}\sim {\mathrm{UPP}}_{4}$ and${\;\mathrm{UPP}}_{1}^{\prime }\sim {\mathrm{UPP}}_{4}^{\prime }$) were employed instead of bilinear interpolation for boosting the surface normal estimation process. The detailed structure of up-projection units is shown in the upper row of Figure 6b.

### 3.4. Refinement Network

For a random pixel i from the coarse depth images, we denote ($h_{i}$, $w_{i}$) as the location of pixel i in 2D space and ($x_{i}$,$\;y_{i}$,$\;d_{i}^{*}$) as the coordinate of corresponding 3D point, where$\;d_{i}^{*}\;$represents the coarse depth value. Similarly, surface normal can be noted as ($n_{x_{i}}$,$\;n_{y_{i}}$,$\;n_{d_{i}^{*}}$). Then, a tangent plane $p_{i}$ [11] can be defined according to Equation (2).

(2) $$n_{x_{i}}\left(x-x_{i}\right)+n_{y_{i}}\left(y-y_{i}\right)+n_{\;d_{i}^{*}}\left(d-d^{*}\right)=0$$

A small 3D neighborhood ($H_{i}$) of i was defined in previous studies [10,38]. For a random pixel $j\in \;H_{i}$, its depth value $d_{j}$. The depth prediction value of pixel i can be computed as Equation (2) according to the pinhole camera model,

(3) $$d_{ji}^{\prime }=\frac{\left(u_{j}-c_{x}\right)n_{x_{i}}/f_{x}+\left(v_{j}-c_{y}\right)n_{y_{i}}/f_{y}+n_{\;d_{i}^{*}}}{\left(u_{i}-c_{x}\right)n_{x_{i}}/f_{x}+\left(v_{i}-c_{y}\right)n_{y_{i}}/f_{y}+n_{\;d_{i}^{*}}}\times d_{j}$$

where $f_{x\;}\;$and $f_{y\;}$ represent the focal length in x and y directions, respectively; $C_{x\;}\;$and$\;C_{y}$ are coordinates of the lens principal points; and K represents the convolution kernel operation. Then, in order to refine depth value of pixel i, we applied kernel regression [10] to operate estimation on all pixels in $H_{i\;}$ as

(4) $$\;d_{i}^{\prime }=\frac{\sum_{j\in \;H_{i}}[d_{j}^{*}K\left(n_{i},n_{j}\right)d_{ji}^{\prime }]}{\sum_{j\in \;H_{i}}[d_{ij}^{\prime }K\left(n_{i},n_{j}\right)]}$$

(5) $$K\left(n_{i},n_{j}\right)=n_{j}^{T}n_{i}$$

where$\;d_{i}^{\prime }$ is the refined depth value $n_{i}$ = [$n_{x_{i}}$,$\;n_{y_{i}}$,$\;n_{d_{i}^{*}}$]. According to the linear kernel algorithm, the angle error between surface normal image *n*_i_ and image *n*_j_ decides whether pixels i and j are in the same tangent plane$\;p_{i}$ or not. Therefore, the smaller angular error contributes to more accurate depth estimation$\;d_{ji}^{\prime }$. 

### 3.5. Loss Function

This section presents loss functions for regression problems existing in depth and surface normal estimation. The loss functions evaluate the difference between the ground truth images and the prediction images generated by the deep learning network. For random pixel i, we define the coarse depth map, refined depth map and ground-truth depth map as$\;d_{i}^{*},\;d_{i}^{\prime }$, and$\;d_{i}^{gt}$ separately. Similarly, we denoted the coarse surface normal map, RGB-D fusion surface normal, and ground-truth surface normal as$\;n_{i}^{*}$, $n_{i}^{F}$, and$\;n_{i}^{gt}$ separately. The total number of pixels is *M*, and the loss function for depth value is defined as Equation (6).

(6) $$l_{depth}=\frac{1}{M}\left(\underset{i}{\sum }{∥d_{i}^{\prime }-d_{i}^{gt}∥}_{2}^{2}+\omega_{1}\underset{i}{\sum }{∥d_{i}^{*}-d_{i}^{gt}∥}_{2}^{2}\right)$$

As shown in Equation (6), the loss function $l_{depth\;}$is the point-wise loss defined according to depth values. On the one hand, $l_{depth\;}$computes the sum of the *L2* norm of the error vectors between coarse depth maps $d_{i}^{*}\;$and ground truth images$\;d_{i}^{gt}$. On the other hand, $l_{depth\;}\;$calculates difference between refined depth maps $d_{i}^{\prime }\;$and ground truth images$\;d_{i}^{gt\;}$over all pixels. The loss for surface normal training process is defined as Equation (7),

(7) $$l_{normal}=\frac{1}{M}\left(\mu_{l}\underset{i}{\sum }{∥n_{i}^{F_{l}}-n_{i}^{gt}∥}_{2}^{2}+\omega_{2}\underset{i}{\sum }{∥n_{i}^{*}\left(t\right)-n_{i}^{gt}∥}_{2}^{2}\right)$$

where l in Equation (7) stands for the scale value [19] of surface normal features and${\;\omega }_{1},$ $\omega_{2}$, and $\mu_{l}\;$stand for weight parameter of loss functions. The loss function $l_{normal\;}\;$computes the *L2* norm of the angular errors of the orientation vectors for each pixel. On the one hand, $l_{normal\;}$computes the *L2* norm of the angular difference between coarse surface normal maps $n_{i}^{*}\;$and ground-truth normal maps$\;n_{i}^{gt}$. On the other hand, $l_{normal\;}\;$calculates the divergence between fusion normal maps $n_{i}^{F_{l}}\;$and ground-truth normal maps$\;n_{i}^{gt}$. There are a lot of step edges such as texture crosshatches or object boundaries [39] in natural RGB images, which inevitably interfere with the accuracy of depth estimation. It is necessary to prevent such interference so as to deal with distorted or blurry problems around step edges. The $l_{grad}$ function defined by Hu et.al. [9] was adopted in this paper in order to penalize depth errors between neighboring pixels of depth images,

(8) $$l_{grad}=\frac{1}{M\;}\overset{n}{\underset{i=1}{\sum }}(F(\nabla_{x}\left(\tau_{i}\right))+F\left(\nabla_{y}\left(\tau_{i}\right)\right))$$

where$\;\tau_{i}={∥d_{i}^{*}-d_{i}^{gt}∥}_{1},\;\;\nabla_{x}\left(\;\tau_{i}\right)$ and$\;\nabla_{x}\left(\;\tau_{i}\right)$ represent spatial derivation of$\;\tau_{i}$ along the *x* and *y* directions, respectively. F(x) stands for a log algorithm function of depth errors defined by Hu [30].

To be concluded, we defined a total loss denoted as$\;l_{total}$

(9) $$l_{total}=\lambda_{1}\;l_{depth}\;+\;\lambda_{2}\;l_{grad}\;+\;\lambda_{3}\;l_{normal}$$

where $l_{depth}$ represents pixel-wise loss for depth values, $l_{grad}\;$represents loss function for step edges, and $\;l_{normal}$ represents function for surface normal.$\;\lambda_{1},$ $\lambda_{2},\;\mathrm{and}\;\lambda_{3}\;$stand for initialed weights working for balancing effects of back propagation.

### 3.6. Recovering 3D Features from Estimated Depth

**Figure 7:**
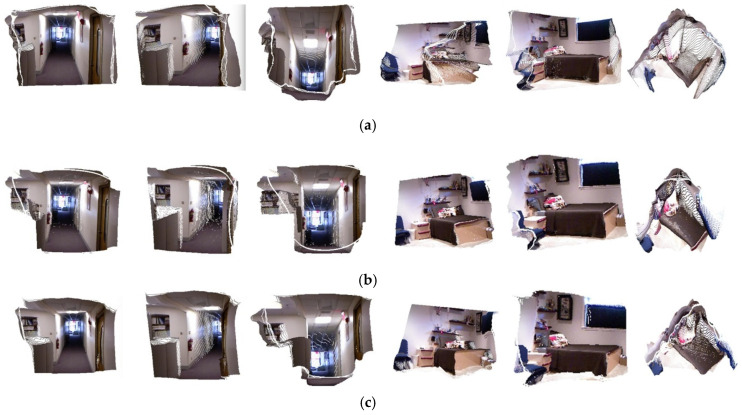
Comparison of point clouds from estimated depth maps between Alhashim [6] and ours. (**a**) Alhashim [6], (**b**) GT, (**c**) Ours. GT stands for 3D point cloud maps from ground truth images.

As is often the case [10,21], 3D space geometric constraints contribute to the capability of deep network in terms of depth estimation and corresponding 3D point cloud reconstruction. Therefore, the 3D point cloud maps shown in Figure 7 were reconstructed following Li et al. [40] to visualize the quality of depth maps predicted by our scheme. 

**Figure 8:**
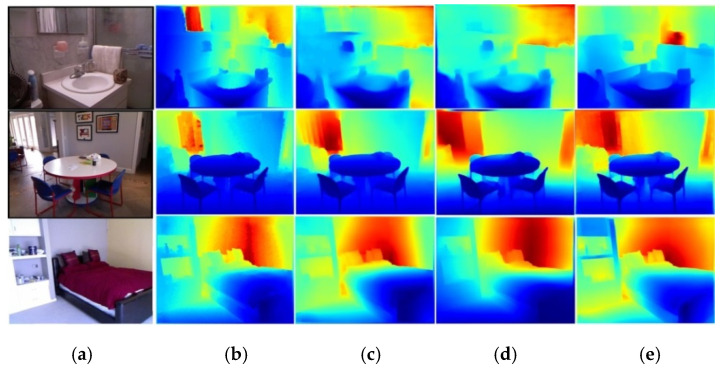
Qualitative results for depth estimation. (**a**) RGB images, (**b**) ground truth (GT), (**c**) Alhashim [2], (**d**) Our Coarse; (**e**) Refined Depth.

We compare the 3D point-cloud maps produced by our scheme based on Densenet-121 with that of Dense-depth [6], which is based on Densenet-169 and does not introduce geometric constrictions. As shown in Figure 7 and Figure 8, our scheme outperforms Dense-depth [6] in terms of depth estimation results and the average quality of the relative 3D point-cloud maps. Therefore, the guidance of surface normal will definitely improve the quality of depth maps in terms of features in 3D space. What is more, better 3D point cloud maps can be recovered from depth maps with constriction of geometric features. As a result, the quality of point-cloud maps should also be adopted as a fundamental metric for evaluating the accuracy of depth estimation.

## 4. Experiments

In this section, we describe the implement details of experiments, evaluate the performance of our depth estimation scheme on NYU-Depth-V2 [14], and compare the prediction results against existing state-of-the-art (SOTA) methods. Moreover, we present results of ablation experiment to analyze the influence of the different parts of our proposed method. 

### 4.1. Dataset

The NYU-Depth-V2 dataset [14] contains 407 K frames taken from 464 different indoor scenes, which were split into 249 training scenes and 215 testing scenes. Specially, 1449 RGB images were accurately labeled with depth images, in which 654 images are annotated for testing phase and others for training phase. All images were collected from videos captured by Kinect RGB-D sensor produced by Microsoft in Redmond, WA, the U.S. NYU-Depth-V2 widely serves as training datasets for supervised monocular depth prediction due to its accurate ground-truth (GT) depth labels and abundant image samples. 

In some previous studies [6,9,10,20], depth estimation networks were trained on subsets sampled from the NYU dataset [14]. In practice [10,20], 30 K training frames were sampled from the raw NYU dataset [14]. The fewer frames used, the better-designed the deep learning schemes [4,6], which outperformed SOTA practices [7,21,41] that were trained on the entire NYU dataset. Moreover, the practice by Hu et al. [9] proved that the models are trained on subsets with more frames performing slightly better, but gains in accuracy did not justify the lower learning efficiency and higher system latency. Therefore, instead of the official splits image set, a subset with 30 K frames was used in this paper. All frames were randomly sampled from 249 training subsets.

The NYU dataset [14] does not provide ground-truth surface normal maps ($N^{GT})$; previous studies [10,21] computed $N^{GT}\;$from in-pained depth images, and thus, the quality of the produced $N^{GT\;}$were up to the in-painting algorithm proposed by Levin [35]. Instead of the in-painting method, the method proposed by Hickson et al. [42] was leveraged for obtaining$\;N^{GT}\;\mathrm{in}\;\mathrm{this}\;\mathrm{paper}$. As for confidence map network, we utilized accurate binary mask images ($I_{M}$) offered by Eigen et al. [21].

### 4.2. Implementation Details

We implemented both the coarse depth estimation (CDE) network and the RGB-D surface normal network (RSN) using the deep learning platform PyTorch by Huang et al. [43] operating on a Tesla M40 GPU produced by NVIDA in Santa Clara, CA, the U.S. with 12 GB capacity. 

The encoder of the CDE network was designed based on the Densenet-121 model, which initializes weights for decoder layers. Specially, the last classification layers of the Densenet-121 model were removed. 

The Adam [44] optimizer was adopted for training the CDE network, and learning rate was set as 0.0001. The raw RGB images with resolution of H×W were down-sampled to H/2×W/2 for boosting training processes and fitting the size of output depth images. We conducted the coarse depth estimation training phase with a batch size of six for 20 epochs. A small subset with 654 samples [14] was employed for testing performance of CDE network, and the batch size for testing phase was set as 32. The network finally produced depth images with resolution of H/2×W/2 and an error evaluation index for supervising the training algorithm. 

In order to avoid interference from over-fitting, four augmentation methods were employed following Cubuk et al. [45] for depth estimation training phase:

(1) The horizontal mirroring operation was applied to both RGB and depth images with a probability of 25%.

(2) Much rotation leads into invalid data for GT depth images [6], so input images in training process were rotated by slight degrees, which ranged from −2 to 2 with a probability of 25%.

(3) Contrast and brightness values of RGB images input were randomly scaled by (0.6, 1.2) with a probability of 50%.

(4) Both RGB and depth images were randomly resized to 320 × 256 with a probability of 50%.

The training process of surface normal was also performed on NYU Depth-V2 [14] and the number of training epochs was also set as 20 with batch size of six. The testing image-set for RSN Network consists of 654 sample RGB images and corresponding ground truth surface normal images produced following the practice by Hickson et al. [42]. For optimizer, the Adam [44] was selected with original learning rate of 1 × 10^−4^, initial parameters $\beta {\;}_{1}\;$= 0.9, $\beta {\;}_{2}$ = 0.999 and weight decay of 1 × 10^−4^. The RSN network also produced surface normal images with the resolution of *H*/2 × *W*/2.

The refinement network functioned as the convolution kernel function [10], which does not demand any training process.

In all training experiments, weight$\;\omega_{1}$ for$\;l_{depth}\;$was set as 0.5 for balancing the importance of coarse depth estimation and depth refinement. Weight$\;\omega_{2}\;\mathrm{for}\;l_{grad}$ was set as$\;\omega_{2}=0.5\;$to balance the influence of two terms. As is often the case [6,7,9,10,20], the hyper-parameters are empirically set as a reasonable value for loss functions. In our study, parameter${\;\omega }_{2}\;$was set as 0.5 according to validations on an officially annotated subset with 1449 images. Weight$\;\mu_{l}\;$for$\;l_{normal}$ was set as$\;\mu_{l}=0.2l,\;$where l = {1,2,3,4}. The parameter l was defined in Section 3.4, which stands for the scale value of images fed to the RSN network.

As shown in Equations (6)–(8), $\;l_{depth}$ calculates the depth value errors for each pixel, $l_{normal}$ calculates angular cosine errors for each pixel, and $l_{grad}$ calculates the gradient errors in the log domain. Therefore, the value of $l_{depth}$ will be obviously bigger than $l_{normal}$ and$\;l_{grad}$. To mitigate this effect, the parameter $\lambda_{1}\;$is set to a small value. According to test results on the annotated small subset, parameters {$\lambda_{1},\;\lambda_{2},\;\lambda_{3}\}\;$were set as reasonable weights { 0.2, 1, 1 }. 

As the proceeding of training process, the model gradually converges. The total number of training parameters for entire network based on Densenet-121 model was approximately 32.4 M. Training was performed for 600 K iterations on NYU Depth-V2 [14], which took 18 h to finish. With repeated inference learning, the value of loss functions$\;l_{grad}$, $l_{normal}$, and$\;l_{depth}$ would converge to zero.

### 4.3. Evaluation Criteria

For quantitatively evaluation, three error metrics were used by the previous work of Eigen et al. [46]: absolute relative error (AbsRel), root mean squared error (*RMSE*), and average ($Log_{10}$) error$\;\left(E_{Log_{10}}\right)$. Moreover, threshold accuracy ($\;T_{re}$) was selected as the accuracy metric. All metrics can be defined as:

(10) $$max\left(\frac{D_{i}^{GT}}{D_{i}^{\prime }},\frac{D_{i}^{\prime }}{D_{i}^{GT}}\right)=\delta <T_{re}$$

(11) $$AbsRel=\frac{1}{S\;}\overset{S}{\underset{i=1}{\sum }}\frac{{\left|D_{i}^{GT}-D_{i}^{\prime }\right|}^{2}}{D_{i}^{*}}$$

(12) $$RMSE=\sqrt{\frac{1}{S}\overset{S}{\underset{i=1}{\sum }}{\left|D_{i}^{GT}-D_{i}^{\prime }\right|}^{2}}$$

(13) $$E_{Log_{10}}=\frac{1}{S}\overset{S}{\underset{i=1}{\sum }}\left|Log_{10}D_{i}^{GT}-Log_{10}D_{i}^{\prime }\right|$$

As is shown in the above metrics, we denote $D_{i}^{GT}$ as the ground truth depth corresponding to pixel i, $D_{i}^{\prime }$ as the relative estimated depth, and S as the total number of pixels with available value in ground truth maps. Here, three different thresholds ($\delta ,\;\delta^{2},\;\delta^{3}$) are set as ($1.25,\;1.25^{2},\;1.25^{3}$) according to conventional works [2,46]. 

Three error metrics [21,27] were used for evaluating surface normal maps in this paper: mean of angle error (*Mean*), medians of angle error (*Median*), and root mean square error (*RMSE*). Moreover, three different thresholds (11.25°, 22.5°, 30°) [21] were used for calculating the specific angular error of pixels.

### 4.4. Benchmark Performance Comparison

**Figure 9:**
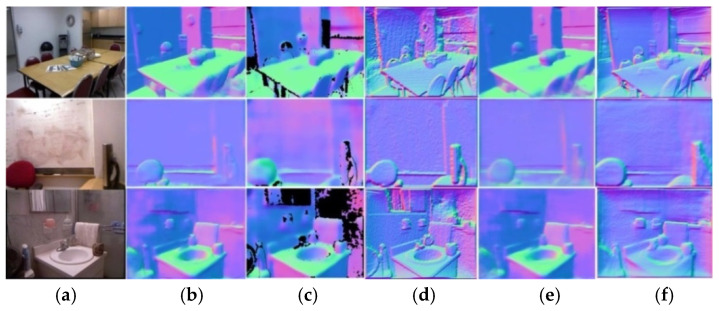
Qualitative results for surface normal estimation. (**a**) RGB images, (**b**) ground truth normal maps, (**c**), Geo-Net [10], (**d**) reconstructed from in-painted ground truth depth (**e**) ours, and (**f**) reconstructed from refined depth. All images are equally scaled for better visualization.

We select the SOTA practice Dense-depth [6] for comparison, which adopted Densenet-169 [5] as the encoder of deep network. The pre-trained model functioned by extracting depth feature from RGB image input. As shown in Figure 8, Dense-depth outperforms the coarse depth estimation network based on Densenet-121 [5] proposed in this paper, while performs worse than our entire depth estimation scheme containing the surface normal guidance. Some depth estimation samples are shown in Figure 9, and more depth maps from our framework are listed in the Appendix A. All images are colorized for better visualization.

Table 1 lists depth estimation results generated by proposed method and previous masterpieces [6,7,9,21]. The quality of depth maps can be evaluated according to Equations (10)–(13).

According to results shown in Table 1 and Figure 8, the refined depth images have a better geometric quality than that of Dense-depth, which proves the necessity of surface normal guidance in monocular depth prediction tasks.

Table 2 compares the surface normal calculation results based on RGB image methods [33,37], surface normal with depth consistency method [10], and the RGB-D fusion method (ours). From Table 2, it can be seen that the surface normal scheme leveraging both depth and RGB image features is averagely superior to the schemes that employ depth images or RGB image only. 

Instead of directly comparing errors between pixel values, Table 2 compares angular difference between orientation vectors for each pixel. The qualitative performance of previous research [10,22,37] is cited from the original papers. As listed in Table 2, with the aid of high-order geometric RGB-D fusion, the surface normal maps from our method outperforms Geo-Net [10]. From Table 2 and Figure 8, we can conclude that the method proposed in this paper can recover better shape from RGB images, which contributes to supply more accurately geometric details with depth images.

### 4.5. Computational Performance

Table 3 compares the computational efficiency of proposed depth estimation algorithm with that of state-of-the-art (SOTA) methods [6,7,9]. It is seen in Table 3 that our model achieves SOTA results on the RMSE metric. What is more, our model requires fewer training parameters, fewer training iterations, and fewer image samples in terms of the same training epochs. Furthermore, our model consumed less training time while operating on similar platforms.

In additional, we tested our pre-trained model and testing models released by references [6,9] on an Tesla M40 produced by NVIDA in Santa Clara, CA, the U.S. with single 12G memory capacity. As shown in Table 3, our model performed lower latency and higher accuracy.

All the data shown in Table 3 were cited from the original paper and corresponding released models [6,9].

### 4.6. Ablation Study

In this section, ablation studies are performed to verify each part of the proposed architecture in terms of performance on depth estimation.

In this experiment, the DenseNet-121 model was substituted with pre-trained DenseNet-161 both in the coarse depth estimation (CDE) network and the RGB-D surface normal (RSN) network. As shown in Table 4, the network based on the DenseNet-161 model [5] outperforms that of the DenseNet-121 model [5] in terms of qualitative accuracy metrics. However, according to the training parameters listed in Table 4, the growth of encoding layers in deep learning deep structure will introduce superfluous training costs.

Furthermore, as shown in Table 4, with the geometric constrictions from surface normal maps, the CDE network based on Densenet-121 model outperforms Dense-depth [6], whose decoder was designed based on deeper-model Densnet-169.

In this experiment, Mobilenet-V2 [47] was adopted as the backbone of depth prediction and surface normal estimation, which utilizes less training weight parameters and lower computational complexity than the Densenet-121 model. As shown in Table 5, the network based on Mobilenet-V2 apparently required less training cost and thus performed lower latency. However, Mobilenet-V2 based deep learning scheme produced worse depth maps than Densenet-121 model [5] at the same time. Therefore, Mobilenet-V2 [47] model-based networks can be conveniently embedded into simple mobile platforms such as a mobile phone and lite drone. The detailed structure of coarse depth estimation network based on Mobilenet-V2 [47] model was shown in Appendix B.

In this experiment, up-projection units in decoder layers of the RGB-D surface normal (RSN) network was substituted with the 2× bilinear up-sampling and up- and down-projection unit [48], respectively, for ablative comparison.

**Figure 10:**
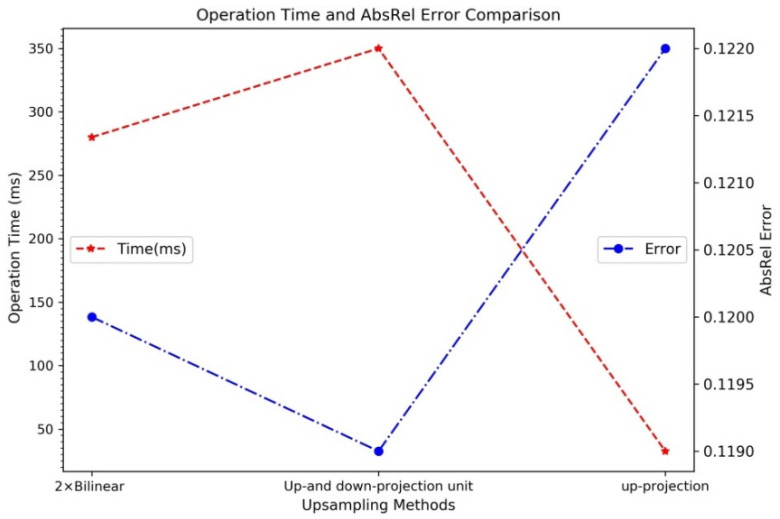
Comparison of time consumption: Runtimes of different up-sampling methods including. 2× bilinear interpolation [6], up and down projection [48], and up-projection [17].

The up-projection unit [17] was designed for embed platforms. As shown in Figure 10, it achieves the lowest latency in this experiment. Therefore, it is suitable to be employed as the up-sampling strategy of the RSN network for boosting surface normal inference process.

The up- and down-projection unit applies super-resolution (SR) techniques to up-scaling the input image to a higher resolution. As shown in Figure 10, the value of absolute relative error (AbsRel) suggests that the refined depth maps obtained by the up and down projection unit slightly outperforms 2× bilinear interpolation [6]. When using the up- and down-projection projection unit [48] in decoder layers, we found that the gains in performance did not justify the slow learning time and the extra GPU memory required. Therefore, the 2× bilinear up-sampling proposed by Alhashimet al. [6] functioned as an up-sampling method in our coarse depth estimation network.

### 4.7. Custom Results

To verify the deep learning network proposed herein, we took some videos of different indoor scenes with the monocular camera of a smart phone. Then, we randomly captured some RGB images from videos and resized them as the resolution of 640 × 480 for depth estimation.

**Figure 11:**
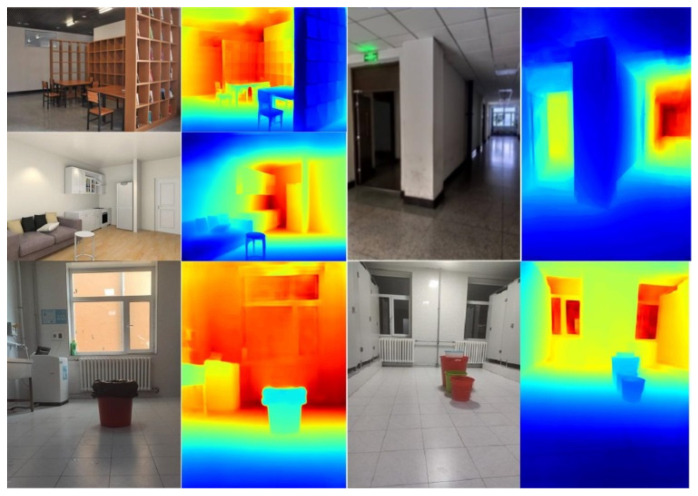
Refined depth images generating from custom images (densenet-161 model).

As shown in Figure 11, depth maps predicted from our network perform distinct boundary and robust geometric shapes.

## 5. Conclusions

In this work, we designed a lighter-weight encoder–decoder deep learning network for depth estimation from monocular RGB images. The encoder layers were designed based on pre-trained deep learning model originally for image classification. Experiments proved that an effective encoder based on transfer learning and geometric guidance outperforms previous methods [7,49] employing complex feature extraction layers. Ablation studies suggested that employing different pre-trained models enabled our networks adapt to different platforms. For example, Mobilenet-V2 [3] can be a suitable model for simple mobile platforms such as smart phones and light micro drones, the Densenet-121 model can be used for mobile platforms equipped with a powerful GPU device, while a denser decoder based on Densenet161 can only be applied to platforms that can afford expensive training costs.

What is more, surface normal estimation was introduced to improve the quality of depth images. Benefiting from geometric guidance offered by surface normal maps, our network obviously achieved better depth images on benchmark NYU depth V2 [14] than state-of-the-art methods [4,7,9]. With the geometric constrictions from surface normal maps, superb 3D point-cloud maps were reconstructed from refined depth images.

Because this work greatly benefits from surface normal estimation, we believe that there are still many other possible geometric features that can be used to improve monocular depth estimation. Therefore, we will further study the effects of geometric features such as 3D objects boundary, semantic label, and image defocus.

Further research will also focus on how to apply pre-trained depth estimation network to 3D vision practices, such as augment reality (AR), simultaneous localization and mapping (SLAM), and structure from motion (SFM).

## Figures and Tables

**Table 1:** Comparison of depth estimation accuracy with previous methods on NYU Depth-V2 [14].

Method	Error (Lower Is Better)			Accuracy (Higher Is Better)		
	AbsREL.	RMSE.	E_Log10_.	δ < 1.25	δ < 1.25^2^	δ < 1.25^3^
Eigen [21]	0.158	0.641	-	0.769	0.950	0.988
Fu [7]	0.115	0.509	0.051	0.828	0.965	0.992
Alhashim [6] (Densenet-169)	0.123	0.465	0.053	0.846	0.974	0.994
Hu [9] (Densenet-161)	0.123	0.544	0.053	0.855	0.972	0.993
Ours (Densenet-121-coarse)	0.137	0.572	0.056	0.839	0.962	0.988
Ours (Densenet-121-refined)	0.122	0.459	0.051	0.859	0.972	0.993

**Table 2:** Comparison of surface normal accuracy with previous methods on NYU Depth-V2 [14].

Method	Error (Lower Is Better)			Accuracy (Higher Is Better)		
	Mean.	Median.	RMSE.	11.25°	22.5°	30°
3DP(MW) [37]	36.3	19.2	46.6	39.2	52.9	57.8
Wang [22]	26.9	14.8	-	42.0	61.2	68.2
Qi [10]	19.0	11.8	26.9	48.4	71.5	79.5
Ours (RGB-D fusion)	20.6	11.0	25.6	47.9	73.2	81.8

**Table 3:** Comparison of computational efficiency and performance on the NYU dataset [14]. The “Parm.’”stands for training parameters, and “Iters.” represents the number of training iterations.

Method	RMSE.	Frames	Epochs	Training Time (h)	Iters.	Inference Time (s)	Parms.
Fu [7]	0.509	120K	-	-	3M	-	110M
Alhashim [6]	0.465	50K	20	20	1M	0.265	42.6M
Hu [9] (Se-Net)	0.530	50K	20	-	1M	0.352	-
Ours	0.459	30K	20	18	600k	0.217	32.4M

**Table 4:** Ablation study of encoder layers. Parm. stands for training parameters; DN stands for Dense-net.

Method	Parm.	Error (Lower Is Better)			Accuracy (Higher Is Better)		
		Abs-REL	RMSE.	E_Log10_.	δ < 1.25	δ < 1.25^2^	δ < 1.25^3^
DN-121-Refined	32.4 M	0.122	0.459	0.051	0.859	0.972	0.993
Dense-depth [2] (DN-169)	42.6 M	0.123	0.465	0.053	0.846	0.974	0.994
DN-161-Refined	67.2 M	0.116	0.446	0.049	0.867	0.976	0.994

**Table 5:** Ablation study of encoder layer numbers. Parm. stands for training parameters.

Method	Parm.	Error (Lower Is Better)			Accuracy (Higher Is Better)		
		AbsREL	RMSE.	E_Log10_.	δ < 1.25	δ < 1.25^2^	δ < 1.25^3^
Ours(121-refined)	32.4 M	0.122	0.459	0.051	0.859	0.972	0.993
Ours(V2-Refined)	4.7 M	0.196	0.519	0.146	0.811	0.951	0.979

**Figure A1:**
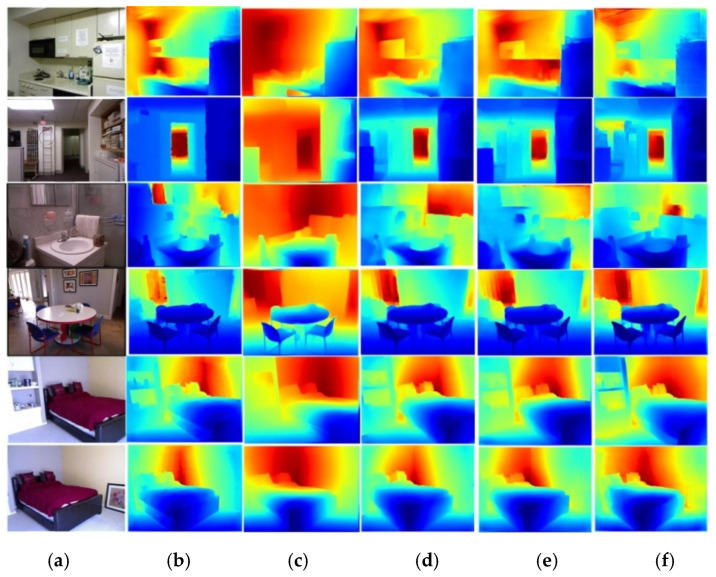
Results of different methods on NYU V2 Depth. (**a**) Red-green-blue (RGB) images, (**b**) ground truth (GT), (**c**) Ranftl [29], (**d**) Hu [30], (**e**) Alhashim [3], (**f**) ours.

**Figure A2:**
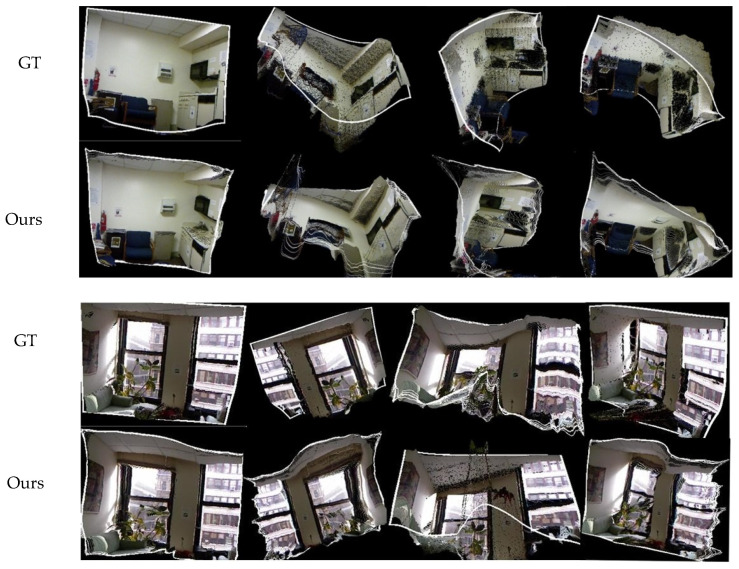
Three-dimensional point cloud maps comparison on NYU Depth V2.

**Figure A3:**
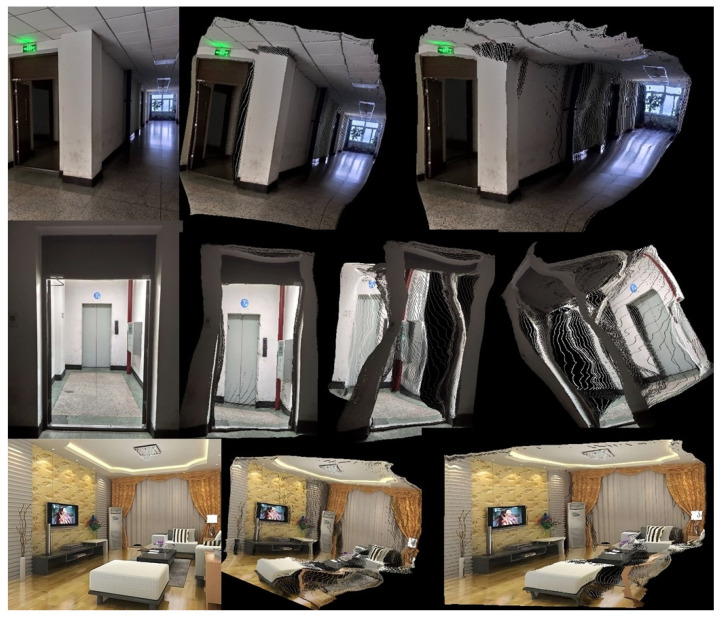
Point cloud maps were reconstructed based on custom images.

## Appendix B

**Table A1:** Details of coarse depth estimation architecture based on Densenet-161.

Layer	Output	Operator
Input	640 × 480 × 3	-
Convolution1 (CONV. 1)	320 × 240 × 96	CONV. 7 × 7
DenseBlock1 [26] (DB.1)	160 × 120 × 96	Avg. Pooling 2 × 2
DB2	80 × 60 × 192	Avg. Pooling 2 × 2
DB3	40 × 30 × 384	Avg. Pooling 2 × 2
DB4	20 × 15 × 1056	Avg. Pooling 2 × 2
CONV. 2	20 × 15 × 2208	CONV. 1 × 1
Bottle Layer	20 × 15 × 1024	Batch-Normal + ReLU + CONV. 1 × 1
UP-Sample1 (US1)	40 × 30 × 1024	Up-sample 2 × 2
Concatenating 1	40 × 30 × 1408	Concatenating DB3 Avg. Pooling
US1-CONV. 1 [2]	40 × 30 × 512	CONV. 3 × 3
US1-CONV. 2	40 × 30 × 512	CONV. 3 × 3
UP-Sample2 (US2)	80 × 60 × 512	Up-sample 2 × 2
Concatenating2	80 × 60 × 704	Concatenating DB2 Avg. Pooling
US2-CONV. 1	80 × 60 × 256	CONV. 3 × 3
US2-CONV. 2	80 × 60 × 256	CONV. 3 × 3
UP-Sample3 (US3)	160 × 120 × 256	Up-sample 2 × 2
Concatenating3	160 × 120 × 352	Concatenating DB1 Avg. Pooling
US3-CONV. 1	160 × 120 × 128	CONV. 3 × 3
UP3-CONV. 2	320 × 240 ×128	CONV. 3 × 3
UP-Sample4 (US4)	320 × 240 × 128	Up-sample 2 × 2
Concatenating4	320 × 240 × 224	Concatenating CONV. 1 Avg. Pooling
US4-CONV. 1	320 × 240 × 64	CONV. 3 × 3
US4-CONV. 2	320 × 240 × 64	CONV. 3 × 3
CONV. 3	320 × 240 ×1	CONV. 3 × 3
Output	240 × 320 × 1	-

**Table A2:** Details of coarse depth estimation architecture based on Mobilenet-V2.

Layer	Output	Operator
INPUT	640 × 480 × 3	-
Convolution1 (CONV.1)	320 × 240 × 32	CONV. 3 × 3
Inverted Residual [3] Block1 (IRB1)	320 × 240 × 16	Bottleneck × 1
Inverted Residual Block2 (IRB2)	160 × 120 × 24	Bottleneck × 6
Inverted Residual Block3 (IRB3)	80 × 60 × 32	Bottleneck × 6
Inverted Residual Block4 (IRB4)	80 × 60 × 64	Bottleneck × 6
Inverted Residual Block5 (IRB5)	40 × 30 × 96	Bottleneck × 6
Inverted Residual Block6 (IRB6)	40 × 30 × 160	Bottleneck × 6
Inverted Residual Block7 (IRB7)	20 × 15 × 320	Bottleneck × 6
CONV. 2	20 × 15 × 1280	CONV. 1 × 1
CONV. 3	20 × 15 × 768	CONV. 1 × 1
UP-Sample1 (US1)	40 × 30 × 768	Up-sample 2 × 2
Concatenating1	40 × 30 × 928	Concatenating IRB6
US1-CONV. 1	40 × 30×384	CONV. 3 × 3
US1-CONV. 2	40 × 30 × 384	CONV. 3 × 3
UP-Sample2 (US2)	80 × 60 × 384	Up-sample 2 × 2
Concatenating2	80 × 60 × 448	Concatenating IRB4
US2-CONV. 1	80 × 60 × 192	CONV. 3 × 3
US2-CONV. 2	80 × 60 × 192	CONV. 3 × 3
UP-Sample3 (US3)	160 × 120 × 192	Up-sample 2 × 2
Concatenating3	160 × 120 × 216	Concatenating IRB2
US3-CONV. 1	160 × 120 × 96	CONV. 3 × 3
UP3-CONV. 2	160 × 120 × 96	CONV. 3 × 3
UP-Sample4 (US4)	320 × 240 × 96	Up-sample 2 × 2
Concatenating4	320 × 240 × 112	Concatenating IRB1
US4-CONV. 1	320 × 240 ×64	CONV. 3 × 3
US4-CONV. 2	320 × 240 × 64	CONV. 3 × 3
CONV. 4	320 × 240 × 1	CONV. 1 × 1
Output	320 × 240 × 1	-
